# Incorporating Immunotherapy with Radiotherapy for Lymphomas

**DOI:** 10.3390/lymphatics1030018

**Published:** 2023-12-07

**Authors:** Paolo Strati, Michael T. Spiotto

**Affiliations:** 1 Department of Lymphoma and Myeloma, Division of Cancer Medicine, The University of Texas MD Anderson Cancer Center, Houston, TX 77030, USA; 2 Department of Radiation Oncology, Division of Radiation Oncology, The University of Texas MD Anderson Cancer Center, Houston, TX 77030, USA

**Keywords:** lymphoma, radiotherapy, immunotherapy

## Abstract

Radiotherapy and/or chemotherapy have been used for nearly 100 years to treat lymphoma. Recently, immunotherapy has been incorporated into the treatment of lymphomas. Here, we will review both the role of immunotherapy in lymphoma as well as the feasibility of incorporating immunotherapies with conventional lymphoma treatments, especially radiotherapy. Immunotherapy agents include checkpoint inhibitors that target the PD-1/PD-L1 axis, CTLA-4, or CD47. In addition, other immunotherapy agents such as bi-specific antibodies and CD19 CAR-T cell therapy are being implemented in various non-Hodgkin’s lymphomas. Extrapolating from observations in other disease sites and incorporating immunotherapy with conventional treatments of lymphoma, including radiotherapy, may have opposing effects. Radiotherapy may stimulate anti-tumor immune responses that synergize with immunotherapies. In contrast, radiotherapy, as well as chemotherapy, may also induce local and systemic immune dysfunction which reduces the efficacy of immunotherapies. With newer radiation treatment techniques and limited radiation fields, it is likely that the efficacy of immunotherapy can be maintained when included with conventional treatments. Therefore, there remains an unmet need to better understand the role of immunotherapy alone and in combination with current treatments in lymphoma patients.

## 1. Introduction

Lymphomas have been treated with a combination of chemotherapy, hematopoietic stem cell transplantation, and/or radiotherapy. Over the last 20 to 30 years, lymphoma was one of the earliest cancers to be treated with a combination of immunotherapy and radiation using B-cell-specific antibodies conjugated to various radioisotopes. However, compared to solid tumors, the treatment of lymphomas has been relatively slow to incorporate other non-radioisotope conjugated immunotherapy agents including checkpoint inhibitors, bispecific antibodies, and adoptive immunotherapy. In contrast, checkpoint inhibitors such as anti-PD1 and anti-CTLA4 have been effective in several solid tumors including melanoma, head and neck cancers, and lung cancer [1,2]. Furthermore, these immunotherapies have been combined with conventional radiation and chemotherapy strategies, which has provided interesting insights into the benefits and limitations of combining immunotherapy with conventional treatment approaches [3–5]. Consequently, understanding how immunotherapy compliments and/or antagonizes radiotherapy and/or chemotherapy in solid tumors may better inform the use of immunotherapy alone or in conjunction with other therapies in lymphoma.

Here, we will describe the emerging role of immunotherapy in lymphomas and how to incorporate immunotherapy with current conventional treatments, especially radiation. We will first describe the role of immunotherapies, including checkpoint inhibitors, bispecific antibodies, and CART cell therapies, that stimulate adaptive immune responses in lymphomas. Next, we will discuss how targeting the lymphoma microenvironment can also elicit anti-tumor immune responses. We will conclude this section by discussing how the molecular subtypes of lymphoma may better dictate how to best incorporate immunotherapies into current treatment strategies. Since these immunotherapies have not been studied with conventional treatments for lymphoma including radiotherapy, we will discuss the opportunities and challenges of incorporating immunotherapies with radiotherapy observed in preclinical models as well as in other cancer sites. Here, we will describe how radiotherapy can both stimulate as well as inhibit the efficacy of immunotherapy by causing systemic lymphopenia as well as immune dysfunction in irradiated lymph nodes. Finally, we will discuss how immunotherapies, namely CART cell therapy, can also induce immune dysfunction. Overall, this review will elucidate the emerging role of immunotherapy in lymphomas and the potential implications of combining immunotherapy with conventional treatments such as radiotherapy.

## 2. Immunotherapy for Lymphoma

### 2.1. Agents That Stimulate Adaptive Anti-Tumor Immune Responses

**Figure 1.**
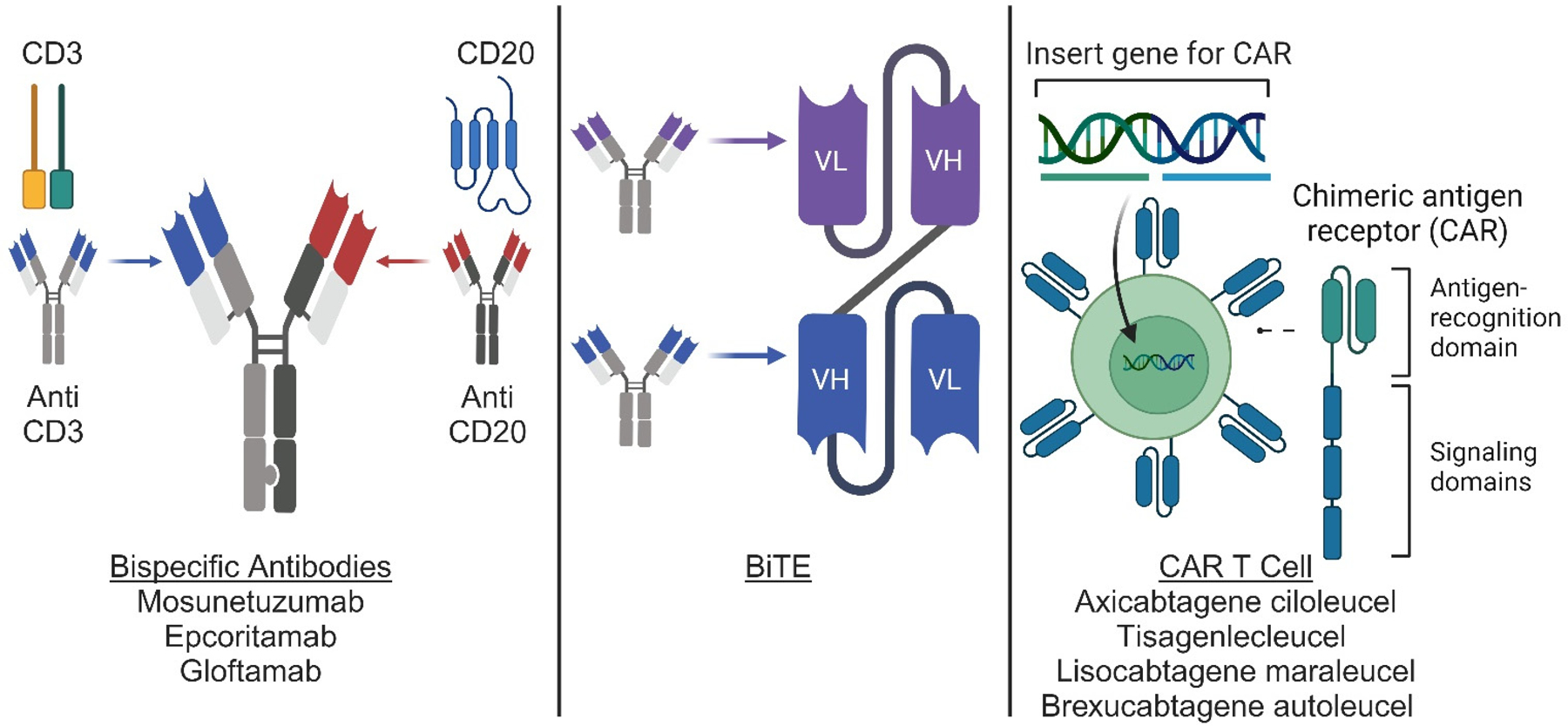
Novel immunotherapy agents for use in lymphoma. Conceptual diagram for bispecific antibodies, BiTEs, and CAR-T cell therapy. Bispecific antibodies (**left panel**) are artificial proteins engineered from the variable chains of 2 different antibodies to generate an antibody capable of recognizing 2 different antigens. BiTEs (**middle panel**) are derived from the variable chains of 2 different antibodies and fused to generate an artificial bi-specific monoclonal antibody. CAR T cells (**right panel**) are derived from T cells engineered through viral transduction to express a modified antigen receptor containing an antigen recognition domain and T cell signaling domains. Illustration produced with BioRender.

Chemotherapy has represented the mainstay of lymphoma treatment for decades, with very limited use of immunotherapeutic agents, mainly represented by cereblon E3 ligase modulatory drugs (such as lenalidomide) and immune checkpoint inhibitors (such as nivolumab and pembrolizumab) [6,7]. However, over the last 5 years, the lymphoma therapeutic armamentarium has been flooded with novel immunotherapeutic agents, with bispecific antibodies (BsAbs) and chimeric antigen receptor (CAR) T-cell therapy (CART) representing the most paradigmatic and impactful examples (Table 1 and Figure 1).

BsAbs are designed to bind two different antigens to bring T-cells in physical proximity to lymphoma cells and favor their killing [8]. Multiple formats have been developed over the years, overcoming technical and clinical challenges, up to recent Food and Drug Administration (FDA) approval. The prototype of BsAbs is represented by bispecific T-cell engagers (or BiTEs), variable fragment (Fv)-based BsAbs, consisting of two single-chain variable fragments (ScFv), joined by a glycine-serine linker, with two binding domains, such as blinatumomab [9]. Thanks to their small size, BiTEs were able to better penetrate the tumoral tissue and activate a T-cell effector response independent of major histocompatibility complex (MHC) restriction. However, this also translated into a short half-life, requiring continuous infusion, and high rates of neurotoxicity [10]. These challenges have been overcome with the development of larger BsAbs that are IgG-like and contain a fragment crystallizable (Fc) region linking the two antibody binding domains [11]. These modifications have allowed for less frequent administration and lower toxicity rates and have recently supported the FDA approval of three anti-CD3/CD20 BsAbs for B-cell lymphoma, including mosunetuzumab for follicular lymphoma (FL) and epcoritamab and glofitamab for large B-cell lymphoma (LBCL) [12–14]. We summarize the efficacy and response rates from pivotal clinical trials in Table 2. We summarize select ongoing clinical trials in Table 3.

CART is instead an adoptive cell therapy with genetically modified T-cells that express a CAR, an engineered receptor with an extracellular tumor binding domain, a transmembrane domain, and an intracellular T-cell signaling domain [15]. Four autologous anti-CD19 CARTs are approved by the FDA for the treatment of lymphoma, including axicabtagene ciloleucel and tisagenlecleucel for FL and LBCL, lisocabtagene maraleucel for LBCL, and brexucabtagene autoleucel for mantle cell lymphoma [16]. Differences in the costimulatory domain have translated into significant differences in CAR T-cell amplification kinetics and associated toxicities. Higher amplification peaks and greater incidence of cytokine release syndrome and immune cell-associated neurotoxicity syndrome are in fact observed with CD28 compared to 4–1BB, impacting the differential use of these products based on patients’ age and comorbid health conditions [17].

Finally, immunomodulatory activity has been observed for other agents that are currently FDA approved for the treatment of lymphoma. These include BTK inhibitors, such as ibrutinib, acalabrutinib, and zanubrutinib, that have been shown to favorably impact the phenotype and function of T-cells and macrophages through BTK/ITK targeting [18,19].

### 2.2. Agents Targeting the LN Microenvironment in Lymphoma

The lymph node microenvironment provides fertile soil for the proliferation of lymphoma cells, through both immune and stromal cells. These include mesenchymal stem/stromal cells, lymphoma-associated macrophages and dendritic cells with a pro-tumoral phenotype, exhausted cytotoxic and CD4+ T cells, regulatory T cells, and natural killer cells [20].

Both nodal exhausted T-cells and extra-nodal non-exhausted T-cells can be engaged and redirected to the CD20+ lymphomatous B-cells with the use of BsAbs, including mosunetuzumab, epcoritamab and glofitamab (see above), the latter through a IFNγ/CXCL10-dependent recruitment mechanism [21,22].

Resident intra-nodal exhausted T-cells can be enhanced with the use of nivolumab and pembrolizumab, immune checkpoint inhibitors currently approved by the FDA for the treatment of Hodgkin lymphoma and primary mediastinal B-cell lymphoma [7]. While historically it has been thought that lymphomatous expression of PD-L1 renders cytotoxic PD1 T-cells already present in the lymph node microenvironment dysfunctional, recent evidence shows that it may also affect initial priming due to constant drainage from adjacent lymph nodes [23]. In addition, PD-L1 can also be expressed on lymph node dendritic cells, and the use of PD-1 inhibitors can help in regulating T cell receptor signaling and CD28 co-stimulation, favoring the activation of tumor-specific T cells, including CD4+ T cells in addition to cytotoxic T-cells [24–26].

Lenalidomide is an oral cereblon E3 ligase modulatory drug currently approved by the FDA for the treatment of indolent B-cell lymphomas and mantle cell lymphoma, but with activity also observed in non-germinal B-center large B-cell lymphoma, including those affecting the central nervous system [6]. As compared to the other immunomodulatory agents outlined above, its impact on the lymph node microenvironment is more pleiotropic. Lenalidomide, in fact, not only favors the reformation of immunological synapses between exhausted T-cells and lymphomatous cells and the activation of natural killer cells but also facilitates the repolarization of lymphoma-associated macrophages to a more anti-tumoral phenotype [27]. In this regard, recent data have shown that macrophage repolarization may be a mechanism of resistance to lenalidomide in B-cell lymphoma, highlighting the potential role of macrophage-targeting agents to improve the efficacy of lenalidomide for this lymphoma subtype [28,29].

While the latter are not currently approved by the FDA for the treatment of lymphoma, some promising agents are being developed. In particular, novel check-point inhibitors able to interrupt the cross-talk between CD47, a don’t eat me tumoral signal, and the macrophage marker SIRPα, such as magrolimab, have shown significant activity in patients with B-cell lymphoma [30]. While their duration of response can be limited when used as single agents, their combination with other agents targeting the lymph node microenvironment is being investigated.

### 2.3. Lymphoma Subtypes That Better Respond to Immunotherapy

The efficacy of immunotherapy in lymphoma may also depend on the subtypes in the involved lymph node and possibly the uninvolved lymph node. Various solid tumors have distinct molecular subtypes that correlate with sensitivity to various treatments including immunotherapy [31]. Toki et al. demonstrated that the uninvolved lymph nodes in patients who failed to respond to immunotherapy for various solid tumors had greater immunosuppressive microenvironments as evidenced by greater numbers of macrophages expressing PD-L1 [32]. Similar subtypes exist in DLBCL, which is divided into five genetic subtypes including: (1) EZB having genetic alterations in the BCL2 and EZH2 locus, (2) BN2 having genetic alterations in BCL6 and NOTCH1, (3) MDC, (4) N1 having mutations in NOTCH1, and (5) primary mediastinal B-cell lymphomas [33]. Of note, primary mediastinal B-cell lymphomas had high rates of durable response with pebrolizumab in the KEYNOTE-013 trial [34]. Similarly, another anti-PD1 inhibitor nivolumab combined with brentuximab vedotin in the CheckMate 436 trial showed high response rates over 70%, with 37% having complete remission [35]. Therefore, the molecular subtypes of lymphomas may help dictate the efficacy of immunotherapy alone or combined with radiotherapy and/or chemotherapy. Further study into the molecular subtypes of lymphomas and their response to immunotherapy may provide novel targets for treatment.

## 3. Challenges to Incorporating Immunotherapy with Radiotherapy

**Figure 2.**
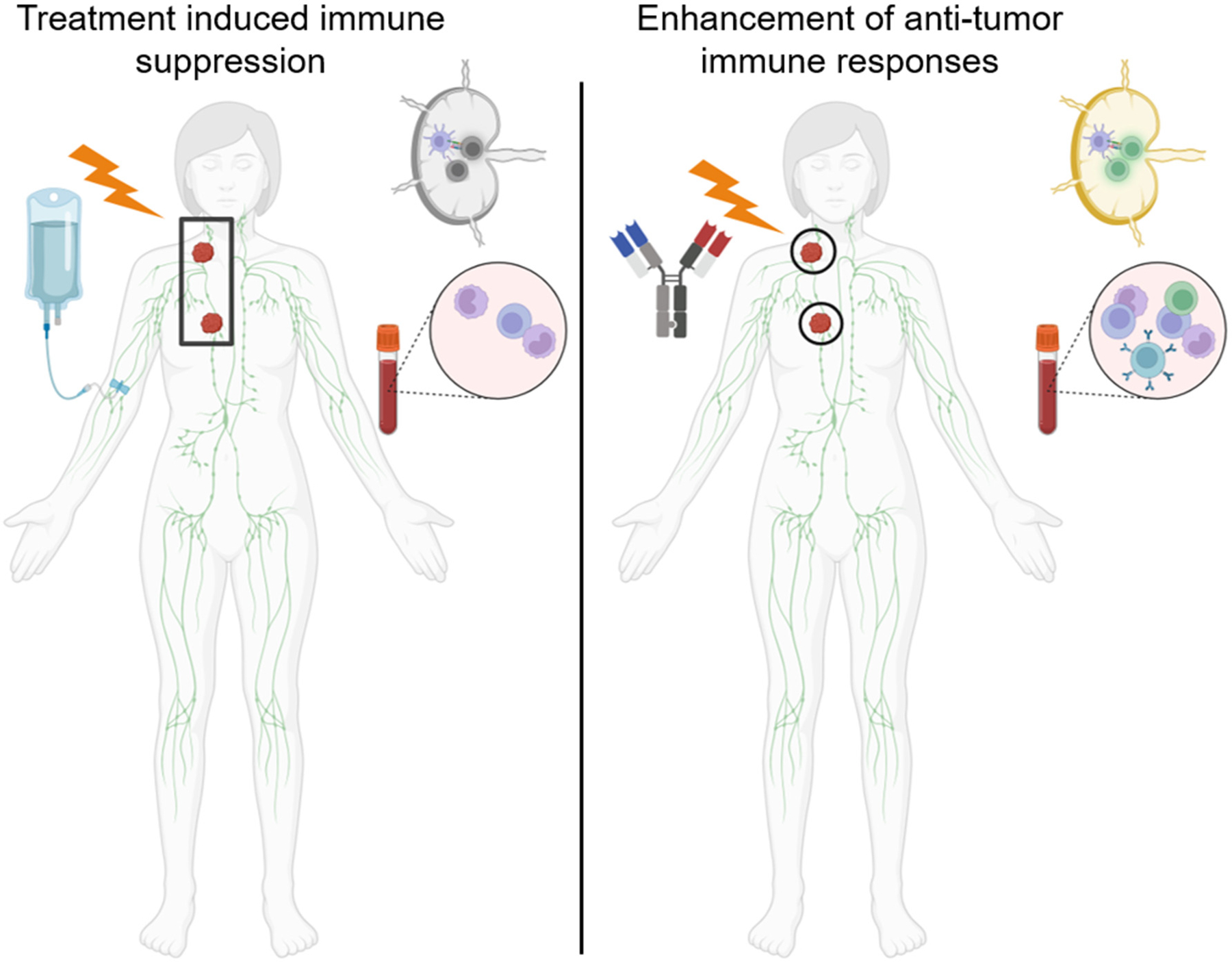
Radiotherapy and chemotherapy can cause lymphatic dysfunction and lymphopenia. (**Left panel**) Radiotherapy encompassing large fields including the tumor draining lymph node and/or chemotherapy can cause immune dysfunction including decreased T cell priming and proliferation as well as systemic lymphopenia which may impair immunotherapy efficacy. (**Right panel**) Treatment with limited-involved-node-type fields may decrease dysfunction in the tumor draining lymph nodes as well as in the systemic immune system to increase the efficacy of immunotherapies.

As immunotherapy has been an emerging treatment modality for both solid and hematological cancers, the incorporation and sequencing of immunotherapies with conventional treatments, in particular radiotherapy, may provide unanticipated challenges and opportunities. Here, we detail some challenges for incorporating radiotherapy with immunotherapy that may better guide the incorporation of immunotherapy with radiotherapy. Since immunotherapy has most often been combined with radiotherapy in solid tumors, we will discuss how radiotherapy in solid tumors has impacted immunotherapy efficacy in order to extrapolate the possible combination of radiotherapy and immunotherapy in hematological malignancies (Figure 2).

### 3.1. Radiation-Related Lymphopenia

Radiotherapy (RT) causes antagonistic immune effects by increasing the immunogenicity of malignant cells while depleting circulating immune cells. RT induces immunogenicity by increasing immunogenic cancer cell death and novel tumor antigen presentation while reducing transient suppression of repressor lymphocyte lineages [36–39]. Several pre-clinical and clinical trials have reported the benefit of immunotherapy with radiotherapy. Lymphomas were the primordial example of combining radiotherapy with immunotherapy with the advent of radioisotopes conjugated to antibodies targeting B-cell epitopes CD19 and CD20 [40–42]. However, the combination of external beam radiotherapy with checkpoint inhibitors or other immunotherapies has been limited. EBRT has been combined with TLR9 agonist to induce systemic immune responses against untreated indolent lymphomas [43]. Furthermore, several groups have established the feasibility of combining anti-PD1 immunotherapy either before, during, or after EBRT in case reports and early stage clinical trials with NK/T cell lymphoma [44] and relapsed/refractory Hodgkin’s lymphoma [45–47]. However, compared to solid tumors, the experience of combining immunotherapies with EBRT is limited. By understanding how radiotherapy and immunotherapy interact, we can better enhance treatments and mitigate the conflicting effects of these modalities.

RT may negatively impact the efficacy of immunotherapy by depleting circulating lymphocytes [48,49] and altering the tumor microenvironment, thereby hampering effective immune responses [50]. This is attributed to radiation-induced lymphopenia and increased regulatory T cell polarization with associated reduced immune responses [51–55]. Both of these phenomena have been associated with worse outcomes and decreased survival [52,54]. In HL, patients treated with extended field radiotherapy developed lymphopenia and decreased reactiveness of lymphocytes in the peripheral blood [56]. Given that radiotherapy fields now focus on the involved site or involved node, there is likely less lymphopenia due to radiotherapy. However, these reduced fields are also combined with chemotherapy which may also cause systemic lymphopenia and/or immune suppression.

To avoid the systemic immune effects of radiotherapy, some groups have partially irradiated tumors in order to stimulate immune responses and avoid radiation-induced immunosuppression [57]. However, partially irradiating cancer is not optimal for patients with curable disease who may then need additional RT if residual disease persists. While emerging studies have employed immunotherapy regimens to stimulate immune responses during radiation, they have demonstrated conflicting results. Preliminary studies incorporating checkpoint inhibitors in both locally advanced cervical cancer and head and neck cancers did not show any improvement in locoregional control or overall survival [4]. This contrasts sharply with the efficacy of combining immunotherapy with CRT for locally advanced lung cancer [5]. One explanation may be that immunotherapy may be more effective when given sequentially after radiotherapy rather than concurrently. Adoptive T cell therapy has been used in leukemia, recurrent cervical cancer, and other solid cancers with promising results on an individual patient basis [58]. However, this approach has not been routinely combined with radiotherapy. Despite multiple emerging immunotherapy strategies to treat both solid tumors and hematological malignancies and cancers, it remains unclear what the best sequencing of immunotherapy with radiotherapy in lymphoma is. One option may be to give immunotherapy after radiotherapy when treating extended nodal regions to minimize the immunosuppressive effects of radiotherapy.

### 3.2. Impact of Nodal XRT on Immunotherapy Effectiveness

Radiotherapy can also cause local immune dysfunction in irradiated lymph nodes and limit the efficacy of immunotherapy. In preclinical models, elective nodal irradiation was associated with immune dysfunction in the tumor DLNs due to increased regulatory T cells and reduced effector T cell polarization which decreased the effectiveness of immunotherapy [31,59–62]. Marciscano et al. used syngeneic melanoma and colorectal tumor models to demonstrate that elective nodal irradiation attenuated chemokine expression, reduced immune infiltration and reduced the effectiveness of anti-CTLA therapy [61]. Buchwald et al. demonstrated that irradiation of only the solid tumor induced CD8+ T cell proliferation in the draining lymph nodes. In contrast, irradiation of the tumor and draining lymph nodes reduced T cell proliferation and the frequency of tumor-specific T cells in a murine melanoma model [59]. Similarly, in head and neck cancer models, ablation of the draining lymphatics either by radiotherapy or surgery negated immunotherapy efficacy [60,62]. These preclinical observations are consistent with immune dysfunction in irradiated pelvic lymph nodes observed in cervical cancer patients [51]. Furthermore, this negative impact of irradiation on draining lymphatics is consistent with the failure of immunotherapy to improve locoregional control or overall survival in cervical and head and neck cancers when given concurrently with RT encompassing elective nodal regions [3,4,63,64]. Consequently, the immune dysfunction associated with ENI likely negatively impacts the efficacy of immunotherapies such as immune checkpoint inhibitors. In lymphoma, nodal irradiation has also been shown to lead to CD8+ T cell dysfunction. Haas et al. demonstrated that nodal irradiation demonstrated a high degree of antigenic immaturity in CD8+ T cells and was cytotoxic to the peripheral blood T cells. Owing to most of the observations regarding immune dysfunction in irradiated lymphoma patients, it may not reflect the immune effects caused by the more conformal involved node or involved site radiotherapy [65]. Given the limited fields and improved radiation delivery for current Hodgkin’s and non-Hodgkin’s lymphomas, the impact of involved nodal therapy may not disrupt the immune system as much as previous involved or extended radiation fields and consequently augment the effects of immunotherapy. Although studies are limited, MacManus et al. demonstrated abscopal regression in 43% of patients treated with low-dose involved-site radiotherapy for indolent NHL which could be augmented with immunotherapy [66]. Consequently, one possibility is that involved nodal RT may stimulate anti-lymphoma immune responses in adjacent non-irradiated lymph nodes. Furthermore, involved field radiation radiotherapy may increase exhausted PD1 + CD8+ T cells’ irradiated field in patients, which could be mitigated with anti-PD1 checkpoint inhibitors [67]. In CAR-T cell therapy, 100% of patients treated with bridging radiotherapy prior to CAR-T transplant demonstrated a response compared to 25% with bridging chemotherapy [68]. Consequently, since chemotherapy is also given with reduced radiation fields, there is still likely a negative impact on the efficacy of immunotherapy for this disease.

### 3.3. CART-Related Lymphopenia

Lymphodepletion is required before the infusion of CART, based on pre-clinical studies showing improved tumor infiltration of adoptively transferred T-cells [69,70]. The favorable impact of lymphodepletion is mediated by multiple biological mechanisms, including but not limited to the elimination of sinks for homeostatic cytokines, mainly interleukin-7 (IL-7) and IL-15, and the eradication of regulatory T cells and myeloid-derived suppressor cells [71–73]. Lymphodepletion is typically achieved with the use of fludarabine and cyclophosphamide, less frequently bendamustine, and it is associated with improved CAR T-cell expansion and persistence as compared to other regimens [73]. Of interest, while patients who receive high-intensity lymphodepleting chemotherapy (LDC) have a higher probability of achieving a favorable cytokine profile, the latter is associated with improved outcome independently of LDC intensity, prompting the question as to whether other immunological factors may play a role [74]. Of interest, in clinical trials where the use of LDC was omitted based on absolute lymphocyte count (ALC) < 1 *×* 109/L before CART infusion, patients experienced worse outcomes, suggesting that pre-LDC lymphocyte count alone may not be sufficient to predict the need for more intensive conditioning regimens and that lymphocyte levels after LDC may also have to be taken into account [75]. In this regard, recent data have shown that the extent of change in lymphocyte count after LDC is associated with worse outcomes after CART, and that variants of genes associated with macrophage biology, such as *MISP* and *CPVL*, affect the latter, suggesting that their manipulation may improve LDC efficacy [76]. Interestingly, the use of bridging therapy has also shown to affect lymphocyte kinetics in response to LDC, emphasizing the need for a biologically rational regimen. The latter may be represented by radiation therapy, a regimen able not only to effectively decrease tumor size but also reduce regulatory T-cells and favorably affect macrophage polarization [77].

LDC-induced lymphopenia can last for several months, beyond the intended duration. In fact, while peripheral CD8+ T cells recover early, CD4+ T-cell recovery is typically delayed, with up to 30% of patients showing a count of <200/mL and 50% of patients showing hypogammaglobulinemia for up to 2 years [78,79]. This translates into infectious complications in up to 40% of patients, including herpes zoster and Pneumocystis jiroveci pneumonia. Of interest, the depth and duration of cytopenia after CART exceed what is typically observed with the use of fludarabine and cyclophosphamide in other settings, suggesting that other immunological mechanisms may be involved [80]. In this regard recent data have shown that CAR T-cell amplification and cytokine production is higher in these patients as compared to those who do not develop cytopenia. In addition, an oligoclonal CD8+ T-cell population expressing interferon gamma has been identified in the bone marrow of these patients, offering a potentially targetable mechanism to limit its complications [41,78,81].

Due to the use of lymphodepleting chemotherapy, patients are at risk of bacterial and fungal infections while neutropenic (typically during the first 30 days after CART infusion), as well as viral and PJP-related infections (up to one year after CART infusion). The severity and frequency of these infections has significantly decreased with the introduction of anti-microbial prophylaxis, including antibacterials and antifungals during the phase of neutropenia, and antivirals and PJP-prophylaxis while CD4 are less than 200 cells/mcL.

## 4. Conclusions

Several clinical reports have illustrated the benefit of immunotherapies in lymphomas. Furthermore, several agents stimulating the adaptive immune response have shown efficacy in lymphomas. Checkpoint inhibitors have been feasible and shown efficacy in both Hodgkin’s lymphoma and NHL. Furthermore, BsAbs, CD19 CART cell therapy, and BTK inhibitors have demonstrated efficacy and gained approval in several NHLs. In addition, they have targeting immunosuppressive mechanisms in the tumor microenvironment, such as anti-CD47, which targets the don’t eat me signal on tumor promoting macrophages. Finally, the molecular profiling of cancers may facilitate better incorporating immunotherapies into the lymphoma subtypes, especially primary mediastinal B-cell lymphomas, which are most responsive to these treatments.

**Figure 3.**
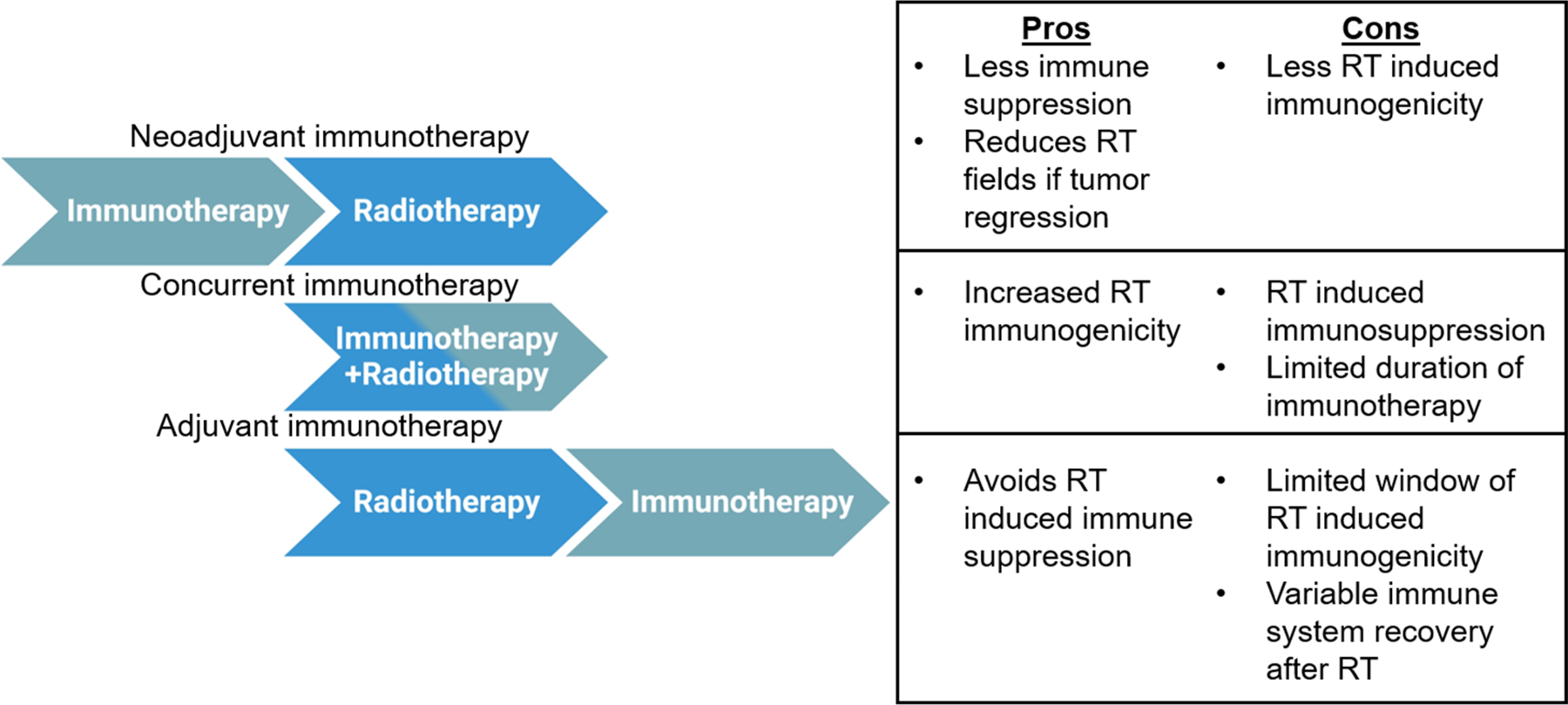
Advantages and disadvantages of various immunotherapy and radiotherapy sequencing strategies. Three basic sequencing strategies may be used with radiotherapy and immunotherapy: neoadjuvant, concurrent, or adjuvant. Pros and cons are given for each strategy.

Since immunotherapies are beginning to be studied in lymphomas, there is an unmet need to better understand how to incorporate these immunotherapies into conventional treatments including radiotherapy. Radiotherapy of the lymph nodes may induce both systemic immune dysfunction, as evidenced by lymphopenia, as well as local immune dysfunction in the lymph nodes that may impair the ability to generate anti-tumor immune responses. This systemic immune dysfunction may be exacerbated by conventional chemotherapies as well as other immunotherapy regimens such as CART cell therapy which also causes lymphopenia. However, modern radiotherapy approaches including involvednode or involved-site radiotherapy as well as the use of image guidance and intensity modulated radiotherapy may minimize these immunosuppressive effects. Consequently, the timing of immunotherapy with radiotherapy may depend on the extent of the radiation field size. For involved nodal radiotherapy, immunotherapy such as checkpoint inhibitors can likely be given concurrently with radiotherapy. However, for larger fields, it may be better to give immunotherapy after radiotherapy to minimize the antagonistic effects of radiotherapy during lymphocyte stimulation. The pros and cons of various sequencings of immunotherapy and radiotherapy are given in Figure 3. For CAR-T cell therapy, radiation is usually effective prior to cellular therapy. Therefore, incorporating immunotherapy into lymphoma will provide another modality to treat this disease as a single agent and possibly in combination with conventional radiotherapy and chemotherapy.

## Figures and Tables

**Table 1.** FDA-approved immunotherapy agents for the treatment of lymphoma.

Agent	Mechanism of Action	Approved Indications
Lenalidomide	Cereblon E3 ligase modulatory drug	Follicular lymphoma Marginal zone lymphoma Mantle cell lymphoma
Nivolumab	PD-1 inhibitor	Hodgkin lymphoma
Pembrolizumab	PD-1 inhibitor	Hodgkin lymphoma Primary mediastinal B-cell lymphoma
Mosunetuzumab	Anti-CD3/CD20 bispecific antibody	Follicular lymphoma
Epcoritamab	Anti-CD3/CD20 bispecific antibody	Large B-cell lymphoma
Glofitamab	Anti-CD3/CD20 bispecific antibody	Large B-cell lymphoma
Axicabtagene ciloleucel	Anti-CD19 autologous CAR T-cell therapy	Follicular lymphoma Large B-cell lymphoma
Tisagenlecleucel	Anti-CD19 autologous CAR T-cell therapy	Follicular lymphoma Large B-cell lymphoma
Lisocabtagene maraleucel	Anti-CD19 autologous CAR T-cell therapy	Large B-cell lymphoma

**Table 2.** Summary of complete response (CR) and overall response (OR) rates.

Agent	Registration Trial	Mechanism of Action	Approved Indication	Response Rate
Mosunetuzumab	II	Anti-CD3-CD20 bispecific antibody	Follicular lymphoma	ORR 80% CR rate 60%
Epcoritamab	I/II	Anti-CD3-CD20 bispecific antibody	Large B-cell lymphoma	ORR 63% CR rate 39%
Glofitamab	II	Anti-CD3/CD20 bispecific antibody	Large B-cell lymphoma	ORR 52% CR rate 39%
Axicabtagene ciloleucel	II (FL) III (LBCL)	Anti-CD19 autologous CAR T-cell therapy	Follicular lymphoma Large B-cell lymphoma	**FL:** ORR 92% CR rate 74% **LBCL (2nd line):** ORR 83% CR rate 65%
Tisagenlecleucel	II	Anti-CD19 autologous CAR T-cell therapy	Follicular lymphoma Large B-cell lymphoma	**FL** ORR 86% CR rate 69% **LBCL (3rd line):** ORR 52% CR rate 40%
Lisocabtagene maraleucel	III	Anti-CD19 autologous CAR T-cell therapy	Large B-cell lymphoma	ORR 86% CR rate 66%

**Table 3.** Ongoing selected trials combining radiotherapy with immunotherapy for lymphoma.

Trial	Phase	Disease	Radiotherapy	Immunotherapy	Status
SIRPant-M (NCT05967416)	I	NHL	2.5 Gy × 3 Involved site	Autologous macrophages	Recruiting
RADVAX (NCT04827862)	II	NHL	4 Gy × 5 involved site	Pembrolizumab (anti-PD1)	Recruiting
MDACC (NCT03210662)	II	NHL	12–22 fractions	Pembrolizumab (anti-PD1)	Recruiting
NCI (NCT04759586)	III	PMBCL	25 fractions	Nivolumab (anti-PD1) with chemotherpay	Recruiting
